# Anti-Jk-b Antibodies and Hemolytic Disease of the Fetus and Newborn: Case-Based Learning

**DOI:** 10.7759/cureus.61948

**Published:** 2024-06-08

**Authors:** Arthi R, Soundharya V, Suresh Kumar I, Hari Haran A, Sahayaraj James

**Affiliations:** 1 Transfusion Medicine, Saveetha Medical College and Hospital, Saveetha Institute of Medical and Technical Sciences, Chennai, IND

**Keywords:** hemolytic disease of fetus and newborn, anti jk-b, antibody identification, antenatal, alloimmunization

## Abstract

The Kidd blood group is clinically significant as Kidd antibodies have the potential to trigger both acute and delayed transfusion reactions, along with hemolytic disease of the fetus and newborn (HDFN). Here, we have reported a case of HDFN due to Jk-b antibodies. A 31-year-old pregnant female was found to have Jk-b antibodies on screening with the Bio‑Rad ID Dia 11-cell panel (Bio-Rad Laboratories, Inc., CA) after her cross-matching results were incompatible. Emergency lower segment caesarian section was done; the baby was non-hydropic at birth with an increase in bilirubin that required high-intensity phototherapy. HDFN resulting from anti-Jk-b incompatibility is rare and tends to present with mild clinical symptoms and a favorable prognosis. However, monitoring of antibody titers is essential to prevent potentially fatal complications. Additionally, antenatal antibody screening should be mandatory for all pregnant women, regardless of their Rh-(D) antigen status, to detect red cell alloimmunization to other clinically significant blood group antigens.

## Introduction

The Kidd blood group system was discovered by Allen et al. in 1951 when an antibody was identified in the sera of Mrs. Kidd whose child suffered from immune hemolytic disease of the fetus and newborn (HDFN). This ‘new’ antibody was named as anti-Jk-a [1]. In 1953, Plaut et al. identified an antithetical antibody anti-Jk-b [2]. Alloantibodies to the Kidd antigens are relatively uncommon [3]. The triggering events are transfusion of blood and blood components, pregnancy and transplantation. However, naturally occurring anti-Jk-b antibodies in a voluntary blood donor have been reported [4].

HDFN is a condition where the maternal antibodies attack fetal red blood cells. These antibodies typically arise from alloimmunization due to incompatible blood groups between the mother and the fetus. Alloimmunization in the mother can result from a transplacental hemorrhage or transfusion. Here, we report a case of anti-Jk-b antibodies causing HDFN.

## Case presentation

A 31-year-old female in her 37 weeks and 4 days of pregnancy with an obstetric score of G2P1L1 came to the emergency department with complaints of decreased fetal movements for the past six hours. She had gestational diabetes mellitus and was on medical nutrition therapy. She had a history of a previous normal vaginal delivery. On vaginal evaluation, she was found to have thick meconium stained liquor. A nonstress test showed fetal distress, and hence, she was taken up for emergency lower segment caesarian section (LSCS).

**Figure 1:**
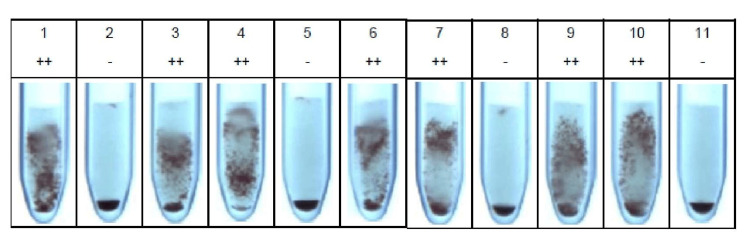
Antibody identification cell panel (Bio-Rad ID-Dia 11-cell panel) showing the cell reactivity pattern with maternal serum

Ethylenediaminetetraacetic acid (EDTA) and clotted blood samples of the patient were sent to our blood center, for blood grouping and typing, and cross‑matching for packed red blood cells (RBCs) before the procedure. Blood grouping and typing were done by column agglutination using Bio‑Rad Diaclon (Bio-Rad Laboratories, Inc., CA, USA). The blood group of the patient was found to be B RhD positive. The cross‑matching procedure was done using a Bio‑Rad column agglutination gel card (Anti‑IgG + Anti‑C3d cards). Cross‑matching was found to be incompatible. The indirect Coombs test was positive indicating the presence of irregular antibodies in the patient’s serum. A detailed immunohematological workup was done. Antibody screening and identification were done using Bio‑Rad ID-DiaCell I, II, III Asia (Mia+), a three-cell panel (lot number 907084.56.2), and Bio‑Rad ID-Dia 11-cell panel (lot number 894233.87.1), respectively. The three-cell panel showed 2+ reactivity in the second cell line. The results of the antibody identification with the Bio‑Rad ID-Dia 11-cell panel are shown in Figure 1.

There was 2+ reactivity in the cell lines 1, 3, 4, 6, 7, 9 and 10 of the Bio‑Rad ID-Dia 11-cell panel. After elucidation by the antigram provided by the manufacturer, anti-Jk-b was identified in the maternal serum. Phenotyping of the mother’s RBC was done and it was found to be Jk (a+b‑). Titration was done for anti-Jk-b antibodies, in the maternal serum with pooled in‑house Jk b + red cell suspension, and was found to be 64 by the semi‑quantitative, doubling dilution method. Phenotyping of the paternal RBC sample was found to be Jk (a-b+).

**Figure 2:**
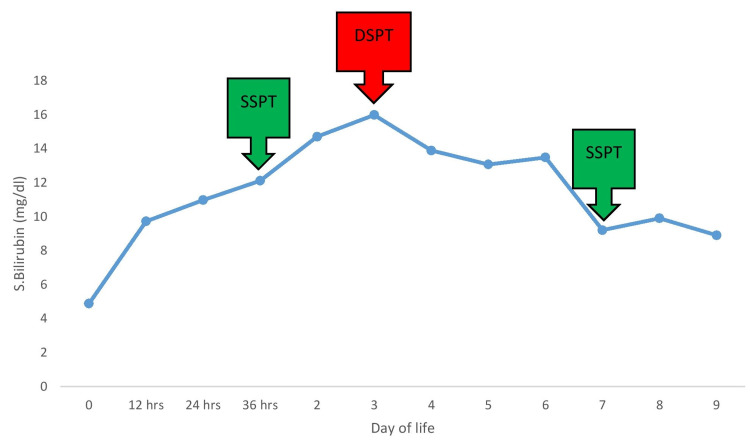
Trend of rise in serum bilirubin and time of intervention SSPT, single surface phototherapy; DSPT, double surface phototherapy

Emergency LSCS was done and a live male baby of 3.31 kg was delivered with an APGAR score of 7/10 at one minute and 9/10 at five minutes. The baby cried after tactile stimulation and was shifted to the NICU in view of respiratory distress with a Downes score of 2/10. On examination, the baby was pale and anicteric and there were no physical signs of hydrops. Cord blood samples were collected and sent for total blood count, serum bilirubin (SBR) analysis, blood grouping and typing, and direct Coombs test (DCT). The blood group of the baby was found to be B RhD positive. DCT was done with the polyspecific (Anti‑IgG + Anti‑C3d) Anti‑human Globulin Coombs gel card and was found to be 2+. Phenotyping of the baby’s RBCs was done and it was found to be Jk (a+b+). An eluate was prepared and antibody identification was done with the Bio‑Rad ID-Dia 11-cell panel (lot number 894233.87.1), and it confirmed the presence of anti-Jk-b. The SBR level was found to be elevated. The trend of rising SBR and time of intervention is depicted in Figure 2.

At birth, the SBR level was found to be 4.88 mg/dl (normal limit, 2-6 mg/dl) and after 24 h of birth, the bilirubin level rose to 9.72 mg/dl. To prevent complications, due to increasing levels of SBR, in the newborn, single surface phototherapy was initiated on day 2 of life. Serial monitoring of SBR was done. The retic count was elevated (8%). Despite being on phototherapy, the SBR levels kept increasing and the baby required double surface phototherapy. Peak SBR was 15.9 mg/dl on day 3 of life. Phototherapy was given for a total of eight days. There were no bilirubin-induced neurological deficits. The baby was shifted to ward on day 10 of life. The baby was gaining weight well and was discharged. The mother was advised to receive Jk-b antigen-negative blood transfusion, if required, even if the antibody screen was negative.

## Discussion

The primary cause of HDFN is often anti‑D alloantibodies, although other blood group antigens can also trigger the condition. The incidence of non‑RhD HDFN has risen in comparison to RhD-related cases since the implementation of anti‑D prophylaxis. Compared to Rh and Kell, Kidd antigens are less immunogenic. The prevalence of Jk-a and Jk-b antigens in the Indian population is 80.8% and 67.8%, respectively [5]. The frequency of Jk (a+b) phenotype in Indians is 29% [6].

Kidd alloantibodies show evanescence and hence have a special importance in the field of transfusion medicine, i.e., despite their initial strong reactivity, they may diminish in detectability within patient serum following a brief period of alloimmunization stimulus [7]. This makes their detection difficult [3]. Alloimmunized individuals should receive lifelong transfusions of antigen-negative blood to prevent an anamnestic response, even if alloantibodies have disappeared [8]. Approximately 52% of Kidd antibodies vanish within months, contrasting with 27% of Rh antibodies [9]. Kidd antibodies also exhibit dosage phenomena, further complicating their detection. Failure to identify them may lead to delayed hemolytic transfusion reactions (DHTRs) or anamnestic transfusion reactions. The prevalence of anti‑Kidd antibodies in the Indian population is 5.9% [3].

Anti-Jk-a and anti-Jk-b are IgG antibodies (incomplete) that form in respective antigen-negative persons following exposure by transfusion or pregnancy (immune antibodies). These antibodies, however, activate complement in a much more efficient way than other IgG antibodies resulting in a greater likelihood of intravascular hemolytic transfusion reaction. The Kidd alloantibodies have the capacity to cause severe acute hemolytic transfusion reaction (AHTR) and DHTR [10]. Case studies have also pointed toward anti-Jk-b as being responsible for severe DHTR [11].

The Kidd antibodies are known to rarely cause HDFN [5]. Kidd antigens can be detected from the seventh week of gestation and are well developed at birth. When alloimmunization occurs, it can potentially lead to HDFN that usually has a mild clinical course and a good outcome [12]. While HDFN caused by anti-Jk-b incompatibility typically presents with mild clinical symptoms with a favorable prognosis, severe anemia and hydrops fetalis can manifest in some cases [12]. Kim and Lee have reported a severe case of HDFN caused by anti-Jk-b requiring exchange transfusion and intensive phototherapy, but the patient died of intractable seizure and acute renal failure on the eighth day of life [13]. Hence, regular monitoring of antibody titers is advisable for all alloimmunized pregnant individuals, coupled with high-risk pregnancy management. An allele-specific polymerase chain reaction (ASPCR) assay can accurately genotype the Kidd blood group system and help in managing pregnancies at risk of Kidd-related HDFN [14].

## Conclusions

This case shows the significance of blood group antigens beyond the Rh system as a potential cause of HDFN. We regard routine antenatal antibody screening as a crucial strategy to be implemented for every pregnant woman, regardless of her Rh (D) phenotype. This proactive approach aims to identify red cell alloimmunization to other clinically significant blood group antigens ensuring comprehensive prenatal care.
